# Analysis of Jmjd6 Cellular Localization and Testing for Its Involvement in Histone Demethylation

**DOI:** 10.1371/journal.pone.0013769

**Published:** 2010-10-29

**Authors:** Phillip Hahn, Ivonne Wegener, Alison Burrells, Jens Böse, Alexander Wolf, Christian Erck, Danica Butler, Christopher J. Schofield, Angelika Böttger, Andreas Lengeling

**Affiliations:** 1 Department of Experimental Mouse Genetics, Helmholtz Centre for Infection Research, Braunschweig, Germany; 2 Department of Biology II, Ludwig-Maximilians-University, Planeeg-Martinsried, Germany; 3 Synaptic Systems GmbH, Göttingen, Germany; 4 Chemistry Research Laboratory and the Oxford Centre for Integrative Systems Biology, University of Oxford, Oxford, United Kingdom; 5 The Roslin Institute and Royal (Dick) School of Veterinary Studies, University of Edinburgh, Roslin, Midlothian, United Kingdom; Tulane University Health Sciences Center, United States of America

## Abstract

**Background:**

Methylation of residues in histone tails is part of a network that regulates gene expression. JmjC domain containing proteins catalyze the oxidative removal of methyl groups on histone lysine residues. Here, we report studies to test the involvement of Jumonji domain-containing protein 6 (Jmjd6) in histone lysine demethylation. Jmjd6 has recently been shown to hydroxylate RNA splicing factors and is known to be essential for the differentiation of multiple tissues and cells during embryogenesis. However, there have been conflicting reports as to whether Jmjd6 is a histone-modifying enzyme.

**Methodology/Principal Findings:**

Immunolocalization studies reveal that Jmjd6 is distributed throughout the nucleoplasm outside of regions containing heterochromatic DNA, with occasional localization in nucleoli. During mitosis, Jmjd6 is excluded from the nucleus and reappears in the telophase of the cell cycle. Western blot analyses confirmed that Jmjd6 forms homo-multimers of different molecular weights in the nucleus and cytoplasm. A comparison of mono-, di-, and tri-methylation states of H3K4, H3K9, H3K27, H3K36, and H4K20 histone residues in wildtype and *Jmjd6*-knockout cells indicate that Jmjd6 is not involved in the demethylation of these histone lysine residues. This is further supported by overexpression of enzymatically active and inactive forms of Jmjd6 and subsequent analysis of histone methylation patterns by immunocytochemistry and western blot analysis. Finally, treatment of cells with RNase A and DNase I indicate that Jmjd6 may preferentially associate with RNA/RNA complexes and less likely with chromatin.

**Conclusions/Significance:**

Taken together, our results provide further evidence that Jmjd6 is unlikely to be involved in histone lysine demethylation. We confirmed that Jmjd6 forms multimers and showed that nuclear localization of the protein involves association with a nucleic acid matrix.

## Introduction

Covalent histone modifications play an important role in regulating chromatin structure, gene expression and epigenetic inheritance [1], [2]. Identified modifications of N-terminal histone tails include phosphorylation, acetylation, ubiquitination and methylation that provide a mechanism for fine-tuned access of the transcriptional machinery to DNA [3]. The various combinations of the histone modifications and the different outcomes on chromatin states, gene expression and consequently on cellular differentiation and development have been collectively referred to as the histone code [4], [5]. The methylation of histone tails is a modification that regulates important processes such as heterochromatin formation, X-chromosome inactivation, imprinting and DNA repair [6]. This posttranslational modification can occur on either lysine or arginine residues in the tails of histones 2, 3, and 4, with lysines being either mono-, di-, or tri-methylated and arginines being mono-methylated or symmetrically or asymmetrically di-methylated [7]. Histone lysine (K) methylation on H3K4, H3K9, H3K27, H3K36, H3K79, and H4K20 has different biological effects. In general [3], [8], methylation on H3K4, H3K36, and H3K79 has an activating effect on gene expression [6], [9], whereas methylation of H3K9, H3K27, and H4K20 impose a repressing effect on transcription [10]–[13].

Until recently, histone methylation was thought to be irreversible. Two types of enzymatic mechanisms for the reversal of histone methylation marks have now been identified. The first histone demethylase identified was the nuclear amine oxidase KDM1 (LSD1), which catalyzes demethylation of H3K4 and H3K9 by employing a flavin dependent reaction [14]–[16]. Members of the Jumonji C (JmjC) domain containing proteins have emerged subsequently and form a second, and apparently larger, family of histone lysyl demethylases [17]–[19]. The JmjC domain of these enzymes contains a double stranded β-helix (or jelly roll) fold which forms a core structure that includes residues that coordinates the Fe(II) and the co-substrate 2-oxoglutarate (2OG). The JmjC demethylases catalyze removal of methyl groups on lysine side chains through an oxidative hydroxylation reaction. The JmjC enzymes are a sub-family of the 2-oxoglutarate and Fe(II) dependent oxygenase superfamily. Bioinformatic analyses suggest that some 2OG oxygenases are highly conserved throughout evolution and many members of this enzyme family can be identified in all living phyla [18], [20], [21]. To date, JmjC-domain-containing histone demethylases (JHDMs [18]) have been characterized that are capable of catalyzing the removal of all methylation states of H3K4, H3K9, H3K36 and H3K27. JHDMs that can demethylate H4K20 and H3K79 have not been identified.

We have been interested in the function of the *Jumonji domain containing gene 6* (*Jmjd6*) [20], [22], [23]. Jmjd6 has been reported to be capable to mediate demethylation of histone 3 arginine 2 (H3R2) and demethylation of histone H4 at arginine 3 (H4R3) [24]. Recently, we have shown that Jmjd6 catalyses the oxidative hydroxylation of the splicing factor U2 small nuclear ribonucleoprotein auxiliary factor 65-kilodalton subunit (U2AF65) and of Luc7-like 2 (LUC7L2) and thereby influences alternative splicing [25]. In contrast to the prior report [24], we did not detect arginine demethylase activity for Jmjd6 [25]. Gene ablation studies of *Jmjd6* in mice and zebrafish have demonstrated its essential role in cell differentiation and development [22], [26]–[28]. *Jmjd6* knockout mice die around birth due to severe cardiopulmonary malformations. These include ventral septal defects, the formation of a double-outlet right ventricle, and hypoplasia of the pulmonary artery that resemble the human congenital syndrome Tetralogy of Fallot [23]. In addition, eye development is severely disturbed in *Jmjd6*-deficient mice ranging from mild retinal defects to ectopic induction of rudimental eye anlagen in nasal cavities [22], [28]. Head, brain and craniofacial dysmorphisms point to defects in the establishment of body plan symmetry [22], [28]. Beside these malformations, differentiation defects are found in the kidney, intestine, liver and lungs during different stages of embryogenesis [22], [27], [28]. Finally, fetal liver derived macrophages differentiated from *Jmjd6* knockout embryos are impaired in the release of pro- and anti-inflammatory cytokines upon stimulation with LPS [22]. Considering the conflicting reports on the biochemical and cellular roles of Jmjd6, the wide ranging defects observed upon *Jmjd6* inactivation in multiple tissues and cells, and the role of other JmjC domain containing proteins in regulating histone methylation, we analyzed if Jmjd6 functions as a JmjC-domain containing histone lysine demethylase.

Using a novel monoclonal antibody for Jmjd6, we investigated its nuclear localization and distribution under various cell culture and growth conditions in different human cell lines and primary mouse embryonic fibroblasts (MEFs). Jmjd6 was found to be located in the nucleoplasma outside of regions containing heterochromatic DNA. This intracellular location was stable and prominent during the interphase of the cell cycle. However, during mitosis Jmjd6 disappears from the nucleus and condensates again in the characteristic fine dotted nuclear pattern of the interphase during the late anaphase and telophase of the cell cycle. Occasionally Jmjd6 was observed in nucleoli indicating that it may be capable of shuttling in and out of this nuclear compartment. Using western blot analysis and immunofluorescence imaging, we compared histone methylation statuses in *Jmjd6* wildtype and knockout cells as well as under Jmjd6 overexpressing conditions. We report that Jmjd6 is not involved in lysine demethylation of H3K4, H3K9, H3K27, H3K36, and H4K20 residues. Instead, we provide evidence that Jmjd6 may preferentially be associated with RNA/RNA complexes which further supports its proposed role in the regulation of RNA splicing.

## Results

### Analysis of intracellular Jmjd6 localization using specific antibodies

**Figure 1:**
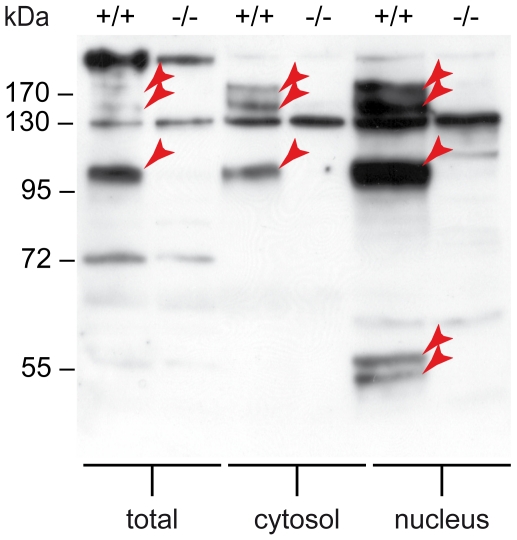
Jmjd6 can be found in multimers in the nucleus and cytoplasm. Western blot showing Jmjd6 protein as detected using antibody AB-10526. Lanes correspond alternating to wildtype (+/+) and *Jmjd6* knockout (−/−) MEF cellular extracts. Total cell extract as well as cytosolic and nuclear extracts have been used (from left to right, respectively). Red arrowheads indicate bands that correspond to Jmjd6.

Several anti-Jmjd6 antibodies are commercially available, however, only one antibody has been validated before in a wildtype/*Jmjd6* knockout context [29]. Initially, we analyzed a set of different anti-Jmjd6 antibodies using western blot analysis. From five anti-Jmjd6 antibodies tested we found two (AB-10526 and AB-11632), which yielded reasonably specific and low background signals when compared on lysates prepared from *Jmjd6* ^−/−^ and *Jmjd6* ^+/+^ embryonic fibroblast cells (Figure S1 and Figure 1). The antibody AB-11632 recognized two fragments of 50 kDa and 55 kDa that are approximately at the calculated size of the mouse Jmjd6 protein (47 kDa), while antibody AB-10562 recognized several fragments of different molecular weights, with four fragments of approximately 50, 100, 150 and 250 kDa being specific for the Jmjd6 protein based on a judgment of staining patterns in *Jmjd6* ^−/−^ and *Jmjd6* ^+/+^ cells (Figure S1 and Figure 1). Previously, western blot and tandem-mass spectrometry analysis have demonstrated that Jmjd6 forms high-molecular weight multimers in the nucleus [29]. To generate a basis for further characterization of Jmjd6 expression, we mapped the binding epitopes of the antibodies AB-10562 and AB-11632. A peptide “spot-membrane” array covering the full-length Jmjd6 protein in 15-mers shifted by three amino acids was generated and hybridized with AB-10562 and AB-11632. The peptide array work revealed that AB-11632 binds to a linear epitope covering amino acids 22–30 at the N-terminal part of the Jmjd6 protein, while AB-10526 recognizes an epitope at the C-terminus covering amino acids 364–372 (Figure S2).

**Figure 2:**
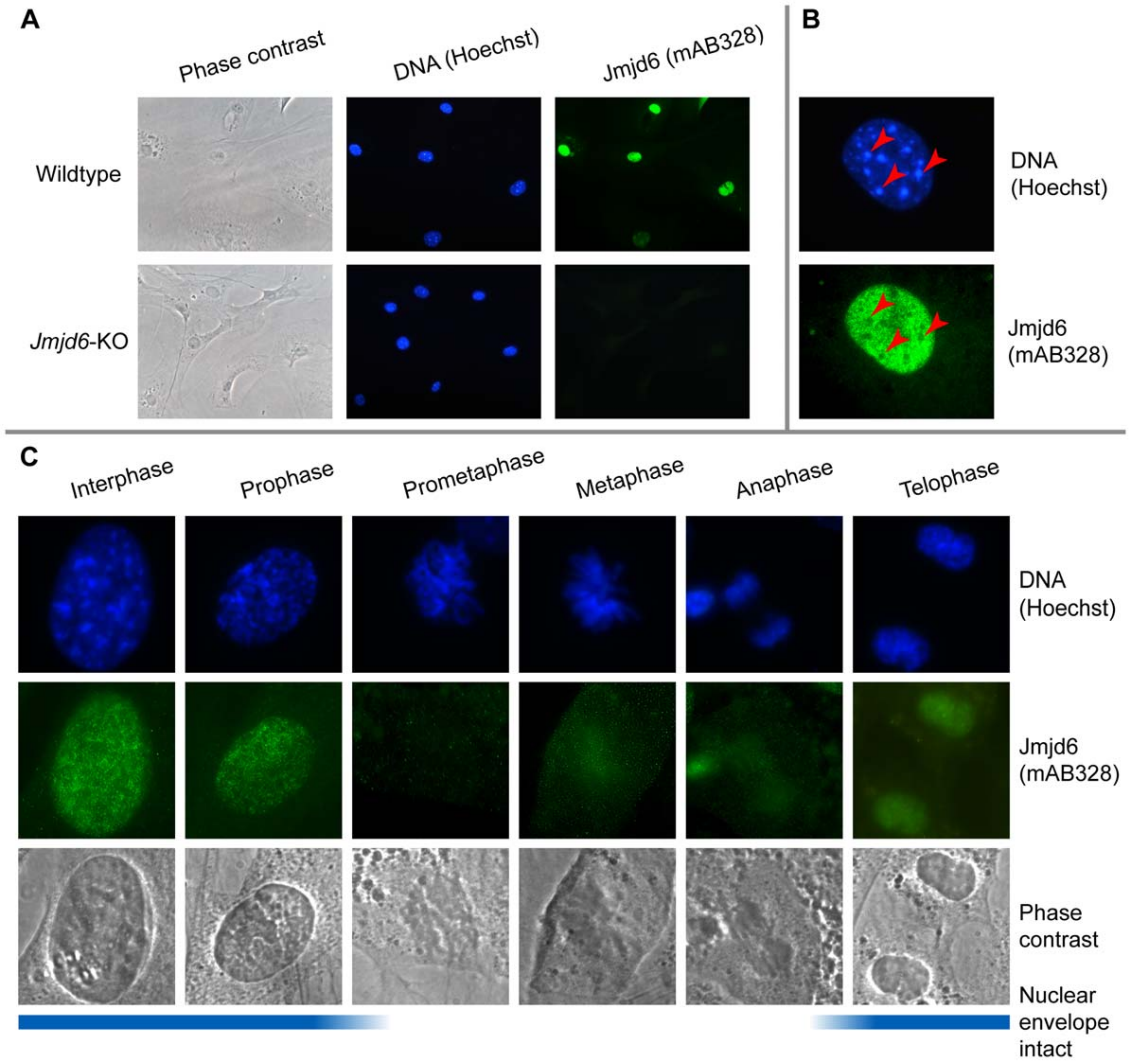
Characterization of Jmjd6 cellular localization using immunocytochemistry and antibody mAB328. (**A**) Immunofluorescence validation of the monoclonal anti-Jmjd6 antibody mAB328. The top row shows immunofluorescence imaging of wildtype, the bottom row of *Jmjd6* knockout mouse embryonic fibroblasts (MEFs). Cells were imaged in phase contrast, with Hoechst DNA stain, and stained using anti-Jmjd6 mAB328 (from left to right, respectively). (**B**) Magnification (100x) of a wildtype MEF nucleus stained with anti-Jmjd6 mAB328. The top image shows wildtype MEF nuclei stained with Hoechst DNA stain (blue), the bottom shows the corresponding nuclei stained for Jmjd6 using mAB328 (green). Red arrowheads exemplify the position of heterochromatic foci. (**C**) Jmjd6 distribution during the cell cycle. Rows show immunofluorescence imaging of stained DNA (blue) and Jmjd6 (green) as well as phase contrast imaging of wildtype MEFs (from top to bottom, respectively). Vertically aligned images correspond to the six cell cycle stages interphase, prophase, prometaphase, metaphase, anaphase, and telophase (from left to right, respectively). The blue line at the bottom indicates the nuclear envelope integrity during the cell cycle.

Because of the difficulties experienced with commercially available antibodies in Jmjd6 detection, we decided to generate a monoclonal antibody specifically for this purpose. Mice were immunized with a recombinant human JMJD6 and 384 hybridoma clones were characterized using cell culture supernatants for immunofluorescence staining of wildtype and *Jmjd6* knockout MEFs. Two out of the 384 selected hybridoma clones showed a differential staining of *Jmjd6* ^+/+^ and *Jmjd6* ^−/−^ cells. These were then further developed using two steps of subcloning, which finally led to the subclone mAB328C6D11 (mAB328) that shows a high specificity for Jmjd6 staining (Figure 2A). The mAB328-staining pattern demonstrated again a nuclear localization of Jmjd6 as previously reported [29]–[33], but it also allowed us to characterize the nuclear distribution of the native Jmjd6 protein in greater detail. In interphase MEFs, Jmjd6 shows a discrete punctual immunofluorescence pattern and is not found in regions of heterochromatic DNA organization as revealed by Hoechst DNA staining (Figure 2B). A similar staining pattern was found in human HEK 293-T cells and in the human lung carcinoma cell line A549 (Figure S3A). Testing mAB328 under denaturizing conditions in western blots and on the spot-membrane showed that this antibody does not detect a linear epitope as no signal could be obtained under various different hybridization conditions (data not shown).

**Figure 3:**
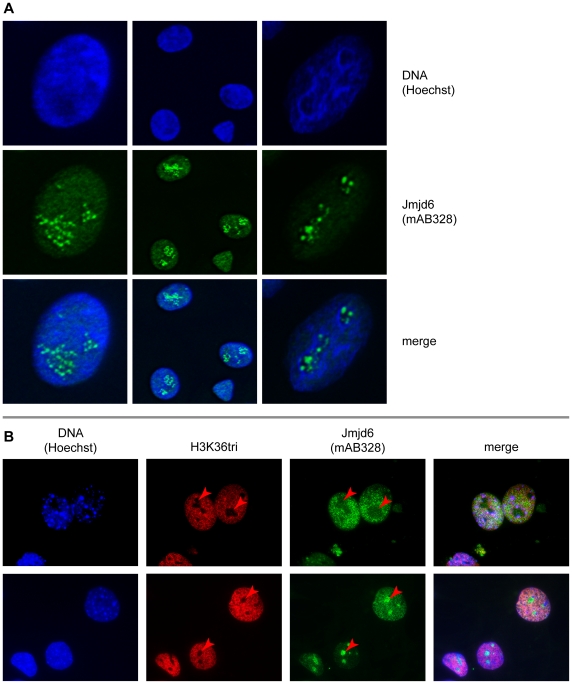
Nucleolar localization and shuttling of Jmjd6. (**A**) Confocal immunofluorescence image of A549 cells with deviant intranuclear distribution of Jmjd6 as compared to its interphase nucleoplasma localization. Horizontal rows show Hoechst DNA stain (blue), Jmjd6 localization (green) and a merge thereof (from top to bottom, respectively). (**B**) Magnification (100x) of wildtype MEF nuclei showing intranuclear Jmjd6 shuttling. Two horizontal rows show immunofluorescence imaging of wildtype MEF nuclei. Images correspond to Hoechst DNA stain (blue), tri-methylated Histone H3 Lysine 36 (H3K36tri, red), Jmjd6 (green), and a merge thereof (from left to right, respectively). Red arrowheads exemplify the position of some nucleoli as indicated by H3K36tri contra-staining.

The distribution of Jmjd6 was found to change throughout the cell cycle (Figure 2C). As soon as the nuclear membrane dissolves during mitosis, Jmjd6 is no longer detected by immunocytochemistry. During interphase, Jmjd6 is distributed in point-shaped clusters in the nucleoplasm as described above. In the prophase Jmjd6 localizes to areas of uncondensed DNA. In the telophase, Jmjd6 staining shows a diffuse pattern with no correlation to DNA (Figure 2C). Arresting cells in the G0-phase using serum starvation or contact inhibition led to a weak and diffuse staining of Jmjd6 (data not shown). After release from G0-arrest, it took 72 h of slow pattern condensation to restore the interphase-staining pattern of Jmjd6 seen before blocking (Figure S3B). Importantly, during these experiments some A549 cells showed a different Jmjd6 distribution pattern as indicated by red arrows in Figure S3B. In these cases, higher magnification revealed a localization of Jmjd6 in small spheres (Figure 3A). Counterstaining with Hoechst dye and comparison to reference images in the Nuclear Protein Database (NPD) [34], implied a nucleolar localization. Staining MEFs with a pan histone H3 antibody as a counter stain showed that different amounts of Jmjd6 were present in nucleoli. In exceptional cases, either nearly all visible Jmjd6 was found in the nucleoli, or the nucleoli were completely devoid of Jmjd6 (Figure 3B). Other nucleoli staining patterns showed intermediate amounts of Jmjd6 in between these two extremes. This indicates that Jmjd6 is capable of shuttling in and out of nucleoli; the factors regulating this shuttling are unknown.

### Characterization of Jmjd6 cellular expression using deletion constructs

Western blots using antibody AB-10526 on cellular fractions of *Jmjd6* ^+/+^ and *Jmjd6* ^−/−^ MEFs confirmed that Jmjd6 (predicted size of ∼47 kDa) is a nuclear protein (Figure 1). Furthermore, as reported, two Jmjd6 polypeptides of ∼50 kDa and ∼55 kDa can be identified only in nuclear fractions [29]. These have been suggested to result from unknown protein modification, possibly involving proteolytic processing of Jmjd6 in the nucleus [29]. In addition, three fragments, at ∼100 kDa, ∼150 kDa, and ∼250 kDa, were observed that further support the previous finding by Tibrewal et al. that Jmjd6 forms homomeric protein complexes in nuclei [29]. These high molecular weight fragments are likely specific for Jmjd6 because they are absent in *Jmjd6* ^−/−^ cells and that they can be found in nuclear and cytosolic cell extracts (Figure 1). Whether the cytosolic complexes in Figure 1 are targeted for proteosomal degradation or serve biological functions remains unclear at this stage.

**Figure 4:**
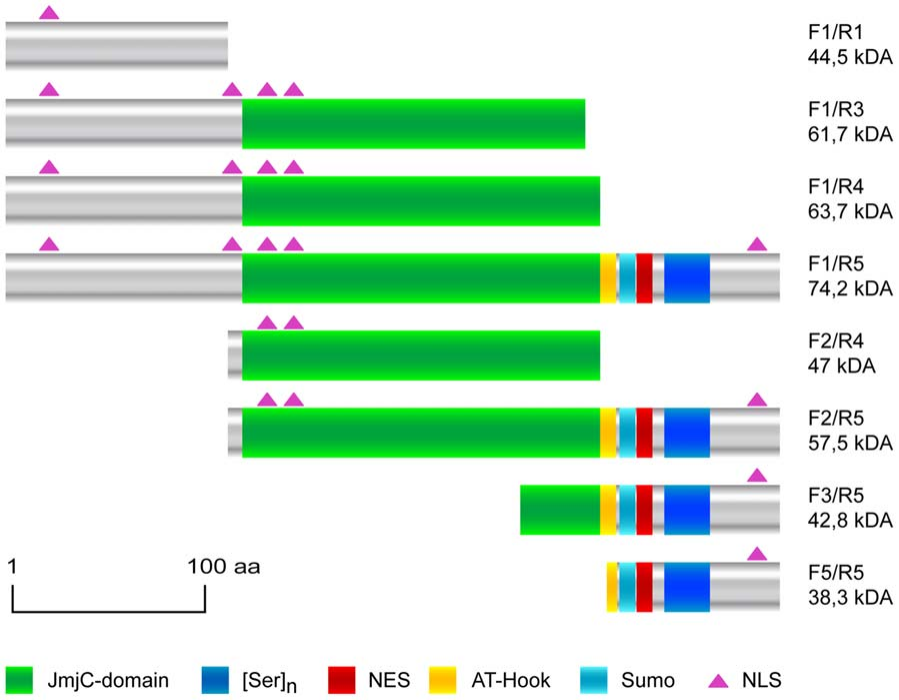
Schematic representation of Jmjd6-YFP deletion constructs. Domains and motifs of the Jmjd6 protein are shown according to the symbols displayed below. The name of each Jmjd6-YFP deletion construct and calculated size when fused to yellow fluorescence protein (YFP, 27 kDa) is indicated on the right side. The scale indicates length of polypeptides in amino acids (aa).

**Figure 5:**
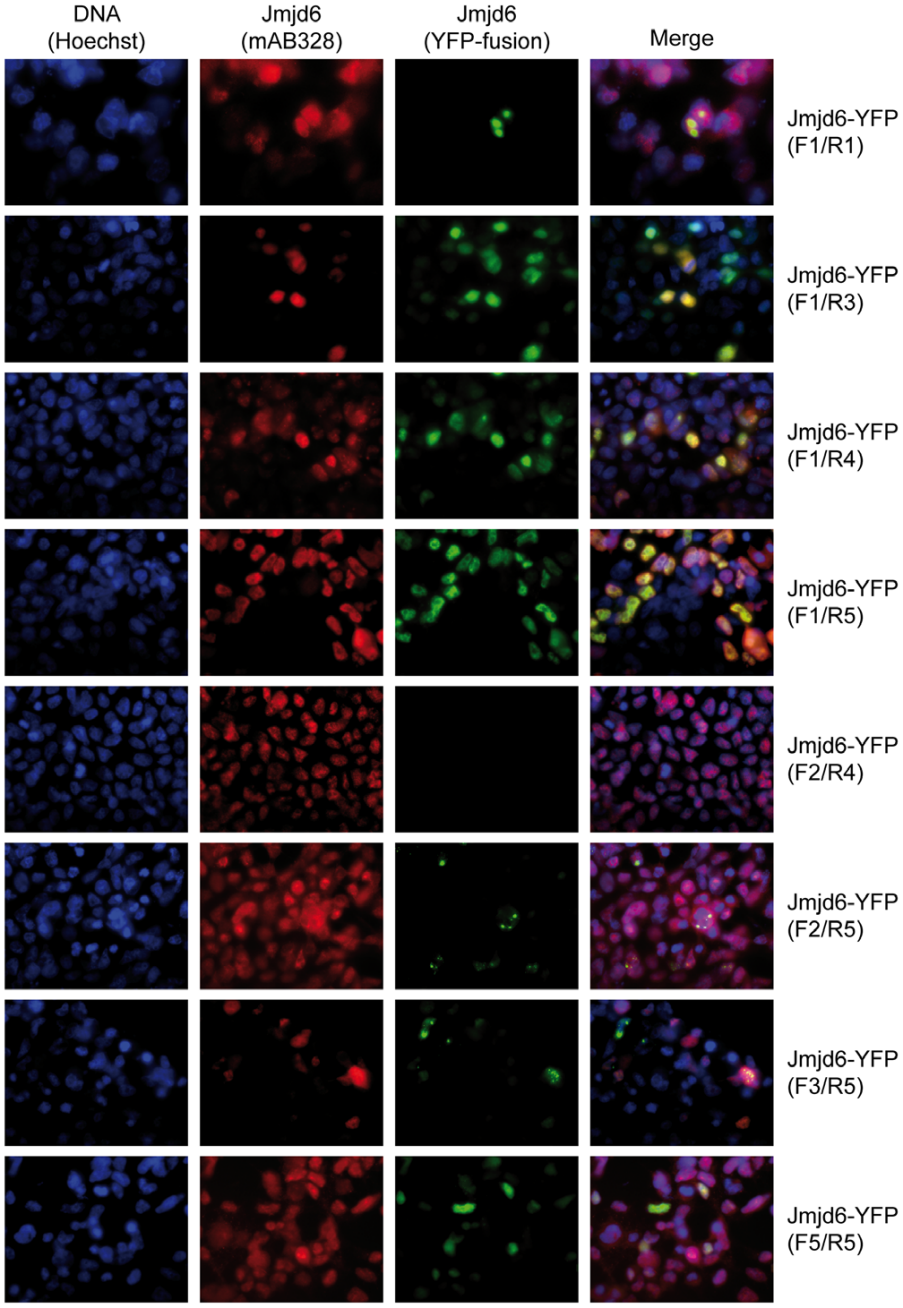
Immunofluorescence imaging of full-length Jmjd6 and deletion constructs fused to YFP. Fluorescent microscopy of Jmjd6-YFP protein fusion constructs transfected into HEK 293-T cells. Horizontal rows correspond to one fusion protein and show localization of Hoechst DNA stain (blue), Jmjd6 (red, stained with mAB328) and Jmjd6-YFP fusion protein as well as a merge thereof (from left to right). Names of Jmjd6-YFP deletion constructs are given in brackets on the right.

In addition to its single double-stranded β-helix domain, Jmjd6 contains a poly-serine stretch and five potential nuclear localization sites (NLS) [31]. Furthermore, an AT-hook [30], a sumoylation site and a nuclear export signal (NES) have been predicted [20]. To analyze the cellular expression of Jmjd6 in more detail, we developed several Jmjd6 deletion constructs, which were fused *C*-terminal to yellow fluorescent protein (YFP) (Figure 4). Beside the full-length protein (F1/R5), three out of the eight constructs contain the N-terminus and are truncated either before the JmjC domain (F1/R1, residues 1 to 126), in the JmjC domain (F1/R3, residues 1 to 288) or immediately after it (F1/R4, residues 1 to 306). One construct contains only the JmjC domain (F2/R4, residues 141 to 291), one contains the complete JmjC domain in addition to the C terminus (F2/R5, residues 141 to 403), and one encompasses the last part of the JmjC domain and the C-terminus of the protein (F3/R5, residues 266 to 403). Finally, construct F5/R5 (residues 306 to 403) contains only the C-terminal part of Jmjd6. The constructs were transfected in HEK 293-T cells and analyzed using fluorescent microscopy (Figure 5). Fusions constructs containing only the JmjC domain (F2/R4) were not detected in cells. Jmjd6 fusion constructs F1/R1, F2/R5, and F3/R5 yielded lower transfection rates in HEK 293-T cells as compared to the other Jmjd6 reporter constructs and the few transfected cells displayed a bright, punctuated expression pattern in proximity to the nucleus. This suggested that these Jmjd6-YFP fusion proteins are probably misexpressed in cytoplasmatic inclusions [35] and are not stable in transfected cells. YFP-fusions containing segments F1/R3, F1/R4, F1/R5, and F5/R5 showed normal localization to the nucleus; comparable to the staining pattern that we observed with the antibody mAB328 (Figure 5).

**Figure 6:**
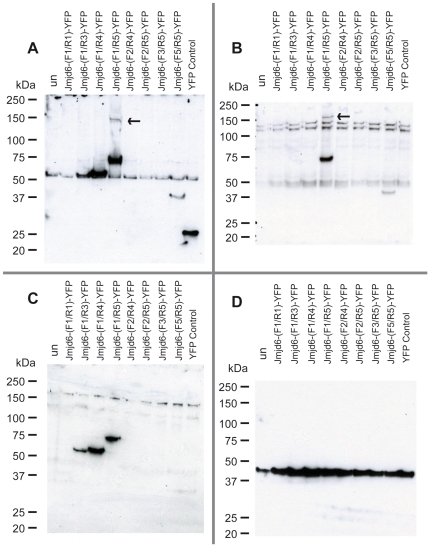
Western blot analysis of nuclear extracts prepared from Jmjd6-YFP fusion construct transfected HEK 293-T cells. YFP fusion constructs from different Jmjd6 deletions were transfected into HEK 293-T cells and nuclear extracts were separated on a SDS-PAGE and blotted. (A) Immunoblot with anti-GFP antibody AB-290 showing expression of Jmjd6-YFP fusion proteins and of a YFP only expressing control in transfected HEK 293-T cells. (B) Immunoblot with anti-Jmjd6 antibody AB-10526 showing expression of Jmjd6-YFP fusion proteins containing the *C*-terminus of the Jmjd6 protein. (C) Immunoblot with antibody AB-11632 recognizing the *N*-terminus of Jmjd6-YFP fusion proteins. (D) Western blot with the anti-β actin antibody AB-6276 serving as a protein loading control for the SDS-PAGE. (un  =  untransfected control, YFP control  =  cells transfected with a YFP expressing control vector, arrows in (A) and (C) indicate expression of a ∼150 kDa fragment corresponding to the size of a Jmjd6-(F1/R5)-YFP dimer).

To further analyze the expression of the Jmjd6-YFP fusion constructs we performed western blot analysis on nuclear extracts of transfected HEK 293-T cells by using the antibodies AB-11632 and AB-10526, which recognize *N*- and *C*-terminal epitopes in Jmjd6, respectively. For detection of YFP fusion tags the anti-green fluorescent protein (GFP) antibody AB-290 was used (Figure 6). Immunoblotting with the anti-GFP antibody revealed expression of the fusion proteins containing the Jmjd6 segments F1/R3, F1/R4, F1/R5, and F5/R5 at the expected fragment sizes (Figure 4 and Figure 6A), demonstrating that these are expressed as monomeric Jmjd6-YFP fusion proteins. A fragment of about 60 kDa was detected in all HEK 293-T cells, including untransfected and YFP-reporter only expressing controls (Figure 6A), indicating that this fragment is unrelated to YFP expression and is recognized by AB-290 non-specifically. Interestingly, only in HEK cells transfected with the full-length fusion protein Jmjd6-(F1/R5)-YFP the antibody AB-290 detected expression of a fragment of about 150 kDa (Figure 6A, arrow). This 150 kDa fragment is expressed at lower levels as compared to the prominent expression of the 74.2 kDa full-length Jmjd6-(F1/R5)-YFP fusion protein. The fragment size of ∼150 kDa suggests that this fragment is most probably a dimer of the Jmjd6-(F1/R5)-YFP fusion protein. Indeed, immunoblotting with the *C*-terminal epitope recognizing antibody AB-10526 showed expression of a corresponding ∼150 kDa fragment only in Jmjd6-(F1/R5)-YFP transfected cells (Figure 6B, arrow). Besides this ∼150 kDa fragment two other fragments of about ∼100 kDa and ∼130 kDA were detected with antibody AB-10526 in all transfected and untransfected HEK cells that are probably representing the endogenous, untagged Jmjd6 dimer (∼100 kDa) and a Jmjd6 unspecific fragment of about 130 kDA that is also present in *Jmjd6*-deficient cells (Figure 1). Both of these fragments were also detectable with antibody AB-10526 on westernblots of MEFs isolated from *Jmjd6* wildtype and *Jmjd6* knockout cells (Figure 1). This suggests that in contrast to all other Jmjd6-YFP fusion proteins only the full-length Jmjd6-(F1/R5)-YFP fusion protein is capable of oligomerisation and dimer formation in transfected cells. A corresponding fragment of ∼150 kDa was not detected with the *N*-terminal epitope recognizing antibody AB11632, suggesting that the epitiope recognized by this antibody is not available for binding in a Jmjd6 dimer (Figure 6C). This is in line with our westernblot results on *Jmjd6* wildtype and *Jmjd6* knockout cells (Figure 1 and Figure S1). Jmjd6-specific high molecular weight fragments indicative of homomultimer formation are only detected with the *C*-terminal epitope-recognizing antibody AB10526 and not with the *N*-terminal epitope binding-antibody AB11632, further suggesting that the *N*-terminal region of the protein is not available for antibody binding in Jmjd6 oligomers. The biochemical nature of Jmjd6 multimerization is still unknown. Tibrewal et al. [29] have suggested that Jmjd6 multimers are formed by covalent cross-linking of protein monomers. Apparently, these Jmjd6 oligomers are not dissociated by separation in denaturating SDS-PAGE gels, however, the underlying mechanism of Jmjd6 multimerization is complex and warrants future investigation into its molecular nature and biological function. Immunoblotting with all three antibodies revealed no expression of Jmjd6-YFP fusion constructs F1/R1, F2/R4, F2/R5, and F3/R5, demonstrating in line with our fluorescent microscopy analysis (Figure 5) that these truncated Jmjd6 proteins are unstable and possibly proteolytically degraded in transfected cells.

### Jmjd6 is not involved in histone lysine demethylation

**Figure 7:**
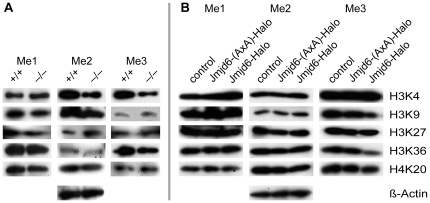
Loss or overexpression of *Jmjd6* does not cause changes in H3K4, H3K9, H3K27, H3K36 and H4K20 histone methylation. (**A**) Immunoblot analysis of *Jmjd6* wildtype and knockout MEFs for differences in histone lysine methylation. All experiments were performed analyzing the mono- (Me1), di- (Me2), or tri-methylated (Me3) forms of histones H3K4, H3K9, H3K27, H3K36, and H4K20 using appropriate antibodies against the modification state of methylated histones. The empty Halo-Tag vector served as control for the expression of Jmjd6-Halo fusion proteins. All lanes contain the same amount of protein and are from the same sample. beta-actin served as a loading control for equal amounts of protein. (**B**) Overexpression of wildtype Jmjd6 or of a Jmjd6 variant with an inactivated JmjC-Domain (Jmjd6-AxA-Halo) both fused to the Halo-Tag in HEK 293-T cells. All experiments were performed analyzing the mono- (Me1), di- (Me2), or tri-methylated (Me3) forms of histones H3K4, H3K9, H3K27, H3K36, and H4K20 as shown in (A). The empty Halo-Tag vector served as control for fusion proteins. beta-actin immunoblotting is shown as a loading control. (A) and (B) represent results from at least three independent experiments.

Recently, studies have identified JmjC-domain containing proteins as histone demethylases that antagonize different methylation states at histone lysine residues H3K4, H3K9, H3K27, H3K36, and H4K20 [19], [36]–[51]. To test if Jmjd6 might be involved in the demethylation of these histone lysine residues, we compared mono- (me1), di- (me2), and tri-(me3) methylation states of H3K4, H3K9, H3K27, H3K36, and H4K20 in MEFs generated from wildtype and *Jmjd6* knockout mice. No differences in methylation states between *Jmjd6* ^+/+^ and *Jmjd6* ^−/−^ MEFs were detected when these histone residues were analyzed using western blots of cellular lysates and specific antibodies (Figure 7A).

**Figure 8:**
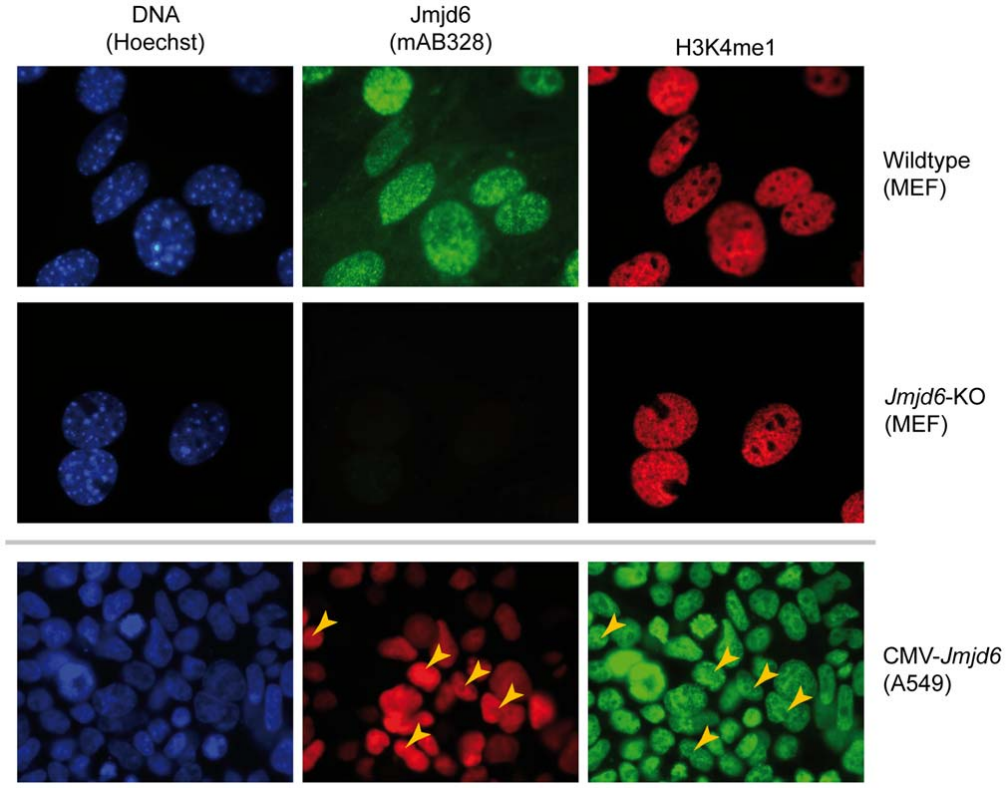
Immunofluorescence analysis of H3K4me1 effects caused by *Jmjd6* deficiency or overexpression. The top two rows show immunofluorescence images of Hoechst DNA stain (blue), Jmjd6 (green), and a histone lysine methylation state specific antibody (red) from wildtype and *Jmjd6*-KO MEFs (from left to right, respectively). Mono-methylated H3K4 was analyzed. The bottom row shows immunofluorescence images of Hoechst DNA stain (blue), Jmjd6 (red), and H3K4me1 (green) in Jmjd6-Halo overexpressing A549 cells (from left to right, respectively). Yellow arrowheads indicate transfected cells. The experiments were performed three times with similar results.

It is established that the Hx[D/E]x_n_H-motif which forms a triad of iron binding residues in the JmjC domain containing proteins is essential for the enzymatic activity of almost all of these proteins [52]. To ensure complete loss of activity [53], we generated Jmjd6-(AxA)-Halo, a Jmjd6 fusion protein variant in which the first histidine and aspartic acid of the catalytical triad are mutated to alanines, thus rendering the JmjC domain of Jmjd6 enzymatically inactive. When overexpressing Jmjd6-Halo or Jmjd6-(AxA)-Halo in HEK 293-T cells, no difference in the amount of methylation of H3K4, H3K9, H3K27, H3K36, and H4K20 between untransfected cells and those overexpressing the different Jmjd6 variants could be detected (Figure 7B). Finally, to verify these findings, single cell analysis was performed on Jmjd6-Halo transfected cells using immunofluorescent imaging. Differences in histone methylation patterns were not detected in cells overexpressing Jmjd6-Halo in comparison to non-transfected controls (Figure 8 and Figures S4, S5 and S6). In addition, histone methylation dependent patterns did not differ in wildtype and *Jmjd6* knockout MEFs when these histone residues were analyzed by immunocytochemistry (Figure 8 and Figures S4, S5 and S6). Taken together, these results demonstrate that Jmjd6 is very likely not directly involved either in mono-, di-, or tri-demethylation of histone lysine residues H3K4, H3K9, H3K27, H3K36, or H4K20.

### Jmjd6 is associated with RNA in cell nuclei

**Figure 9:**
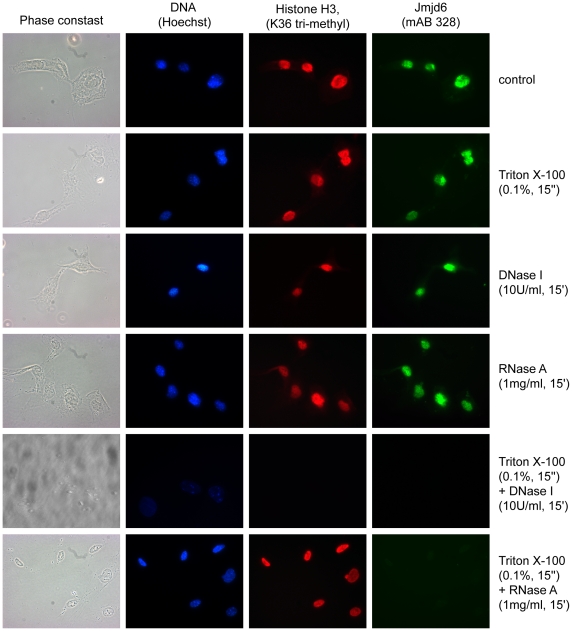
For nuclear localization of Jmjd6 an intact ribonuclear matrix is needed. Wildtype MEFs were treated with Trition X-100, RNase A, DNase I or a combination of Trition X-100 with RNase A or DNase I before fixation and staining. Time intervals and concentration of reagents used in the experiments are indicated on the right side. Untreated cells were used as controls. Horizontal rows correspond from left to right to a phase contrast view and immunofluorescence imaging of Hoechst DNA stain (blue), H3K36tri (red) and Jmjd6 (green). Shown is one representative result of at least three experiments performed.

Cikala and colleagues proposed that Jmjd6 might possess an AT-hook DNA binding domain just C-terminal of the JmjC domain, which might allow binding of the protein to DNA [30] though structural predictions suggest maintaining a functional AT-hook whilst maintaining catalytic function is unlikely. To investigate this hypothesis, we treated MEFs (Figure 9) and HEK 293-T cells with Triton X-100 and with DNase I and RNase A and used mAB328 for Jmjd6 immunofluorescence staining. Treating cells only with Triton X-100 before fixation did not show any effect on Jmjd6 nuclear staining pattern. This observation implies that Jmjd6 is not a highly mobile protein, possibly because it is associated with some kind of nuclear matrix or complex, which prevents the protein from being washed out of perforated cells. Incubation of cells with Triton X-100 and DNase I before fixation and mAB328-staining removed Jmjd6 and DNA completely, indicating that this matrix has an important effect on Jmjd6 localization. To investigate a possible association of Jmjd6 with RNA, we performed a combination of Triton X-100 and RNase A incubation before fixation. This resulted in a complete removal of Jmjd6 from the cells but left the DNA intact. This indicates that Jmjd6 might be associated with RNA and/or RNA complexes and is washed out of cells as soon as the nuclear matrix is lost, e.g. by DNA degradation.

## Discussion

We have investigated the expression of Jmjd6 in nuclear compartments and analyzed regions of the protein required for its nuclear expression. We also tested for a possible role of Jmjd6 in histone lysine demethylation and analyzed a potential association of the protein with nucleic acid/nucleic acid complexes.

The key findings of our work are as follows. First, we show that the endogenous Jmjd6 protein is located in punctuated clusters in the nucleoplasm of human and mouse cells during the interphase of the cell cycle. This discrete punctuated expression pattern is lost when cells start to divide and the nuclear membrane dissolves. During the interphase of the cell cycle Jmjd6 is occasionally observed in nucleoli suggesting that it is capable of shuttling in and out of nucleoli. Previously, we, and others, have reported [22], [29]–[33] that Jmjd6 is a nuclear protein. These results were based primarily on transfection studies with overexpressing Jmjd6-reporter constructs fused to protein tags because of the unavailability of suitable antibodies for immunocytochemistry. In this study, we investigated the expression of the endogenous Jmjd6 protein using immunocytochemistry and the novel monoclonal antibody mAB328, which was specifically generated for this purpose and validated for specific recognition of Jmjd6 using *Jmjd6* knockout cells. Second, we show that Jmjd6 is likely not involved in histone lysine demethylation of H3K4, H3K9, H3K27, H3K36, and H4K20 residues, at least under natural conditions.

### Nuclear expression of Jmjd6

Jmjd6 contains five potential nuclear localization signals [31] and one recently predicted nuclear export signal [20]. Using the monoclonal antibody mAB328 we found Jmjd6 expressed in the nucleoplasm of interphase cells outside of regions containing heterochromatic DNA. Interestingly, Jmjd6 was also found to be localized in nucleoli. The attendant circumstances of this localization could not be experimentally dissected. Different intensities observed in Jmjd6 nucleoli staining patterns suggest that Jmjd6 can shuttle between the nucleoplasm and these subnuclear compartments. Nucleoli are highly dynamic structures and the nucleolar proteome can vary to a great extend under different metabolic and cellular growth conditions [54]. In addition, recent studies have shown that nucleoli play a major role in the physiological adaptation of cells to exogenous stress such as DNA damage, heat shock or hypoxia [55]–[57]. Thus, it may be that Jmjd6 is involved in the regulation of cellular stress responses in nucleoli. Treatment of cells with RNase A and DNase I is providing evidence that the nuclear localization of Jmjd6 is dependent on an intact ribonuclear matrix in the nucleoplasm. As soon as RNA is degraded, the interphase-staining patterns of Jmjd6 is lost (Figure 9), thus implying that association with RNA causes nuclear concentration of Jmjd6. Mechanisms underlying these interactions of Jmjd6 with RNA remain to be established, but may involve the binding of Jmjd6 to splicing regulatory proteins or direct binding to RNA [25]. In fact, support for a direct interaction of Jmjd6 with RNA has recently been published. By using band shift assays Hong and colleagues found that Jmjd6 binds to single stranded RNA [58]. If this interactions relates to the nuclear localisation of the protein or to its catalytic activity remains open and will require future studies regarding its specificity.

In line with the study by Tibrewal et al we found further evidence for expression of Jmjd6 multimers in nuclear and cytoplasmatic extracts (Figure 1). Westernblot analysis of HEK 293-T cells transfected with Jmjd6-YFP fusion constructs detected expression of a fragment most likely corresponding to an YFP-tagged dimer form of Jmjd6. The crystal structure of the Jmjd6 protein has recently been solved [58], [59]. Jmjd6 possesses the characteristic double-stranded β-helix (DSBH) fold that forms the catalytic centre of the enzyme, which has been described for all known 2OG oxygenase structures [58], [59]. Mantri and colleagues have demonstrated that recombinant Jmjd6 protein oligomerises in solution and forms a stable complex as a homodimer [59]. Elucidation of the Jmjd6 dimer crystal structure revealed a complex interaction between two monomomers. The interface is composed of interactions between *N*- and *C*-terminal α-helixes and of loops between α-helixes and β-sheets of both monomers [59]. These regions of interactions are located at the *N*- and *C*-terminal parts of both molecules and explain why we could detect expression of a fragment corresponding to the size of a Jmjd6-YFP dimer only in cells transfected with the full-length Jmjd6-(F1/R5)-YFP fusion construct (Figure 6). All other truncated Jmjd6-YFP fusion proteins were either not properly expressed in transfected cells or lacked critical regions for intermolecular interactions between monomers. Future studies will address if interaction between Jmjd6 monomers *in vivo* are solely formed by strong electrostatic interactions or if further posttranslational modifications are required for covalent linkage of monomeric molecules and Jmjd6 multimer assembly.

### Jmjd6 is not a JmjC-domain containing histone lysine demethylase

Jmjd6 was reported to be capable of catalysing demethylation of histone 3 arginine 2 (H3R2) and of histone H4 at arginine 3 (H4R3) [24]. These data were based on assays involving *in vitro* substrates and recombinant JMJD6 protein as well as on studies overexpressing Jmjd6-reporter fusion proteins in HeLa cells [24]. In our studies reported here, Jmjd6 was not implicated in histone lysine demethylation of H3K4me3, H3K9me2, H4K20me2 and of H3K36 methylated *in vitro* with the Set2 methyltransferase [24]. We demonstrate in this work using comparison of global methylation states in wildtype and *Jmjd6*-deficient MEFs that Jmjd6 is not involved in catalysing demethylation of mono-, di-, and tri-methylation states of H3K4, H3K9, H3K27, H3K36, and H4K20 in cells at least under natural conditions. This is further confirmed by analysis of A549 and HEK 293-T cells transfected with Halo-tagged wildtype and enzymatically inactive Jmjd6 reporter constructs using western blot analysis (Figure 7) and by immunocytochemistry assays with antibodies suitable to detect all histone lysine methylation states (Figure 8 and Figures S5, S6 and S7). Although we have not tested for all possible demethylation substrates (e.g. H3K79), together, with the results of Webby et al. [25] on a lack of lysine demethylation, our results imply that Jmjd6 is unlikely to be involved in histone lysine demethylation.

Most recently, we identified Jmjd6 to interact with splicing regulatory proteins and to modify some of these by posttranslational hydroxylation [25]. This work also did not find evidence for arginine demethylation activity by Jmjd6. Instead Jmjd6 was found to catalyse lysyl-5-hydroxylation of the splicing factor U2AF65 *in vivo*, and of LUC7L2 *in vitro* [25]. Hydroxylation of these proteins by Jmjd6 changed alternative splicing [25]. By treating cells with RNase we found that nuclear Jmjd6 might be preferentially associated with RNA. However, further studies are needed to investigate if a direct interaction of Jmjd6 with RNA *in vivo* is needed for nuclear localization and function.

## Materials and Methods

### Chemicals, enzymes, medium and cells

Chemicals and restriction enzymes were from Sigma (St. Louis, USA) and New England Biolabs (Ipswich, USA), respectively. DMEM cell culture medium was from GIBCO (Carlsbad, USA), FCS Gold foetal calf serum from PAA Laboratories (Pasching, Austria). The human A549 (DSMZ, No. ACC 107) and HEK 293-T (DSMZ, No. ACC 305) cell lines were obtained from the German Collection of Microorganisms and Cell Cultures (DSMZ, Braunschweig, Germany). For preperation of nuclear cell extracts from transfected HEK 293-T cells the NE-PER nuclear and cytoplasmatic extraction reagents have been used together with the Thermo Scientific Halt Protease Inhibitor Cocktail (Thermo Scientific, Pierce Biotechnology, Rockford, USA). Standard cell culture conditions were used for A549 and HEK 293-T cell lines, harvesting and cell culture of mouse embryonic fibroblasts (MEFs) was described previously [60]. Cells where either arrested in G_0_ through serum starvation or contact inhibition.

### Expression constructs and transfection

Sequences of primers used for the cloning of Jmjd6 fusion, reporter and deletion constructs are given in Table S1. Jmjd6-Halo fusion constructs were obtained by cloning full-length Jmjd6 cDNA (Clone IRAV D0197p968D6, ImaGenes, Berlin, Germany) amplified with primer pair 1 into pHT2 (Promega, Madison, USA) using *Bam*HI/*Nhe*I. The Jmjd6-(AxA) variant containing an inactivated JmjC-domain was obtained by replacing the JmjC domain and C-terminal part of Jmjd6 using the product of primer pair 2. The forward primer contains two mutations to inactivate the HxD motif of Jmjd6 (HID changed to AIA in the predicted catalytical triad). The PCR product was digested with *PfI*MI and *Xho*I and exchanged against the same fragment of Jmjd6, gaining full-length Jmjd6-(AxA). A YFP expression vector was generated by amplifying full-length YFP with primer pair 3 and cloning it into pCMV-Tag1 (Stratagene, La Jolla, USA) using *Nhe*I/*Xho*I. Jmjd6-YFP deletion constructs were obtained by amplifying parts of Jmjd6 from clone IRAV D0197p968D6 using primer pairs 4 to 11 and cloning them into the YFP expression vector using *Not*I/*Sal*I. Correct reading frames of all constructs were confirmed by sequencing before usage.

Cells and cell lines were transfected with these constructs using Nanofectin (PAA Laboratories, Pasching, Austria) or using FuGENE HD transfection reagent (Roche Diagnostics Ltd., Burgess Hill, UK) according to the manufacturers instructions.

### Monoclonal antibody generation and epitope mapping

Generation of a monoclonal antibody against Jmjd6 was performed as described [61] using human recombinant JMJD6 for immunization. Initial testing of hybridoma cells used cultivation supernatant in immunofluorescence imaging of wildtype and *Jmjd6-*knockout MEFs. Positive antibody producing cells were further selected in two rounds of subcloning.

Epitope mapping was performed on a spot-membrane covering Jmjd6 in fifteenmers, each shifted by three amino acids. The membrane was blocked for 1 h in skim milk and incubated with the primary antibody in Genosys blocking buffer (Sigma-Genosys, St.Louis, USA) 1∶10 in TBS over night according to manufactures instructions. The membrane was washed 2×20 min in TBS-T (0,1% Tween in TBS) and incubated for 1 h with a horseradish peroxidase (HRP) conjugated secondary antibody in Genosys blocking buffer. After washing the membrane twice in TBS-T, it was imaged with ECL plus (GE Healthcare Life Sciences, Uppsala, Sweden) for chemifluorescence.

### Immunofluorescence staining and imaging

Antibodies used for immunocytochemistry are listed in Table S2. Cells growing on cover slides were washed in TBS, fixed with 4% para-formaldehyde on ice for 15 min and washed once with TBS before blocking with block-sperm buffer (1% BSA, 0.1% Triton X-100 in TBS) for 1 h. After washing once with TBS, cells were incubated with the primary antibody for 1 h in antibody buffer (1% BSA in TBS). Cells were washed three times with TBS and incubated with the appropriate secondary antibody and Hoechst DNA stain (1∶200) in antibody buffer for 1 h. After washing three times with TBS, cells were imaged using fluorescence microscopy with a Zeiss Axiovert 100 microscope using a Plan-Neofluar 100x/1.3 NA oil immersion lens. A digital AxioCam HRc camera (Zeiss, Göttingen, Germany) was used for documentation. All experiments were performed at least in triplicate. For histone methylation state analysis, we examined about 150 cells in each experiment.

For RNase A or DNase I treatment, cells were incubated in 0.1% Triton X-100 for 15 sec, washed twice and incubated with RNase A (1 mg/ml in PBS, Invitrogen) or DNase I (10 U/ml in PBS, Roche, Indianapolis, USA) for 15 min and washed twice with PBS prior to fixation.

### Cell fractioning, immunoprecipitation and western blotting

Cells were washed in PBS, scrapped in Lysisbuffer (10 mM Hepes, 1,5 mM MgCl_2_, 10 mM KCl, 0.2% NP-40, 0.2% 2-mercaptoethanol, proteasome inhibitor, and kept on ice for 20 min. After centrifuging at 2000 g for 5 min, the cytosolic fraction was collected as supernatant. The pellets were resuspended in Nucleusbuffer (20 mM Hepes, 1,5 mM MgCl_2_, 400 mM NaCl, 25% Glycerol, 1 mM DTT, proteasome inhibitor), kept on ice for 20 min and centrifuged again (2000 g for 5 min). The supernatant was collected as nuclear fraction. Nuclear extracts were prepared from transfected HEK 293-T cells using the NE-PER reagents from Pierce Biotechnology (Rockford, USA) according to manufactures instructions.

SDS-Page separated samples were blotted on nylon membranes (Immobilon, Millipore, Billerica, USA) for 25 min at 100 V using blotting buffer (0.3% Tris, 1.44% Glycin, 20% MeOH, 0.375% SDS. The membrane was washed in TBS-T, blocked in Western milk (0,1% Tween in skim milk) for 1 h and incubated with the first antibody in Western milk over night. After washing twice with TBS-T for 20 min, the membrane was incubated with an appropriate HRP conjugated secondary antibody in Western milk, washed again (2×20 min in TBS-T) and imaged using ECL plus for chemifluorescence. Experiments were performed in triplicate.

## Supporting Information

Figure S1 Western blot analysis of wildtype and Jmjd6 knockout MEFs using different anti-Jmjd6 antibodies. This panel shows western blots with total cell lysates from wildtype and Jmjd6-KO MEFs using four different antibodies against Jmjd6. AB-32740, AB-28349, AB-28348, and AB-11632 were evaluated. Red arrowheads mark bands not present in Jmjd6-knockout lanes. All lanes contain the same amount of protein and are from the same sample. Beta-actin served as a loading control for equal amounts of protein on the gel.
(1.57 MB PDF)

Figure S2 Epitope-mapping of AB-11632 and AB-10526. A spot membrane covering the full length Jmjd6 protein in fifteenmers, each shifted by three amino acids, was incubated with AB-11632 and AB-10526. (A) AB-11632 binds to seven fifteenmers and six thereof show high intensity. The core epitope covers the amino acids DWTRHN. (B) AB-10526 binds to seven fifteenmers and five thereof show high intensity. The core epitope covers the amino acids GDG.
(0.07 MB PDF)

Figure S3 Jmjd6 staining pattern in different cell lines and Jmjd6 distribution over time after releasing A549 cells from a G0-block. (A) Comparison of Jmjd6 nuclear expression pattern between murine MEFs and the human cell lines A549 and HEK 293-T. Cells were stained with Hoechst DNA stain (left panel) and for Jmjd6 using the anti-Jmjd6 mAB328 antibody (right panel). (B) Vertical image pairs correspond to a time point 6 h, 24 h, 48 h, or 72 h after releasing A549 cells from a G0-block. The large images show Jmjd6 distribution in green, the small insets the corresponding Hoechst DNA stain in blue. Red arrowheads indicate nuclei with deviant intranuclear Jmjd6 distribution. Shown is one representative experiments of at least at least three performed.
(4.22 MB PDF)

Figure S4 Single cell analysis of H3K9 and H4K20 methylation effects caused by Jmjd6 deficiency or overexpression. The top two rows show immunofluorescence images of Hoechst DNA stain (blue), Jmjd6 (green), and a histone lysine methylation state specific antibody (red) from wildtype and Jmjd6-KO MEFs (from left to right, respectively). The bottom row shows immunofluorescence images of Hoechst DNA stain (blue), Jmjd6 (red), and a histone lysine methylation state specific antibody (green) in Jmjd6-Halo overexpressing A549 cells (from left to right, respectively). Yellow arrowheads indicate transfected cells. Figure (A) shows results for H3K9me1, (B) for H3K9me2, (C) for H4K20me1, and (D) for H4K20me3. All experiments shown were performed at least at least three times with similar results.
(0.76 MB PDF)

Figure S5 Immunofluorescence analysis of H3K4 and H3K36 methylation effects caused by Jmjd6 deficiency or overexpression. The top two rows show immunofluorescence images of Hoechst DNA stain (blue), Jmjd6 (green), and a histone lysine methylation state specific antibody (red) from wildtype and Jmjd6-KO MEFs (from left to right, respectively). The bottom row shows immunofluorescence images of Hoechst DNA stain (blue), Jmjd6 (red), and a histone lysine methylation state specific antibody (green) in Jmjd6-Halo overexpressing A549 cells (from left to right, respectively). Yellow arrowheads indicate transfected cells. Figure (A) shows results for H3K4me1, (B) for H3K4me2, (C) for H3K4me3, (D) for H3K36me1, (E) for H3K36me2, and (F) for H3K36me3. The experiments were performed at least three times with similar results.
(1.22 MB PDF)

Figure S6 Immunofluorescence analysis of H3K27 methylation effects caused by Jmjd6 deficiency or overexpression. The top two rows show immunofluorescence images of Hoechst DNA stain (blue), Jmjd6 (green), and a histone lysine methylation state specific antibody (red) from wildtype and Jmjd6-KO MEFs (from left to right, respectively). The bottom row shows immunofluorescence images of Hoechst DNA stain (blue), Jmjd6 (red), and a histone lysine methylation state specific antibody (green) in Jmjd6-Halo overexpressing HEK 293-T cells (from left to right, respectively). Yellow arrowheads indicate transfected cells. Figure (A) shows results for H3K27me1, (B) for H3K27me2, and (C) for H3K27me3. The experiments were performed three times with similar results.
(0.85 MB PDF)

Table S1 Primer pairs used for cloning of Jmjd6 fusion/reporter proteins. Sequences of primers used for the cloning of Jmjd6 fusion, reporter and deletion constructs.
(0.04 MB PDF)

Table S2 Antibodies used in Western blotting and immunofluorescence imaging. List of antibodies used for western blotting and immunocytochemistry.
(0.06 MB PDF)
