# Supplementary figures and images for: Analysis of Jmjd6 Cellular Localization and Testing for Its Involvement in Histone Demethylation
Source: PLoS One. 2010 Oct 29;5(10):e13769. doi: 10.1371/journal.pone.0013769 (PMC2966431; doi:10.1371/journal.pone.0013769)

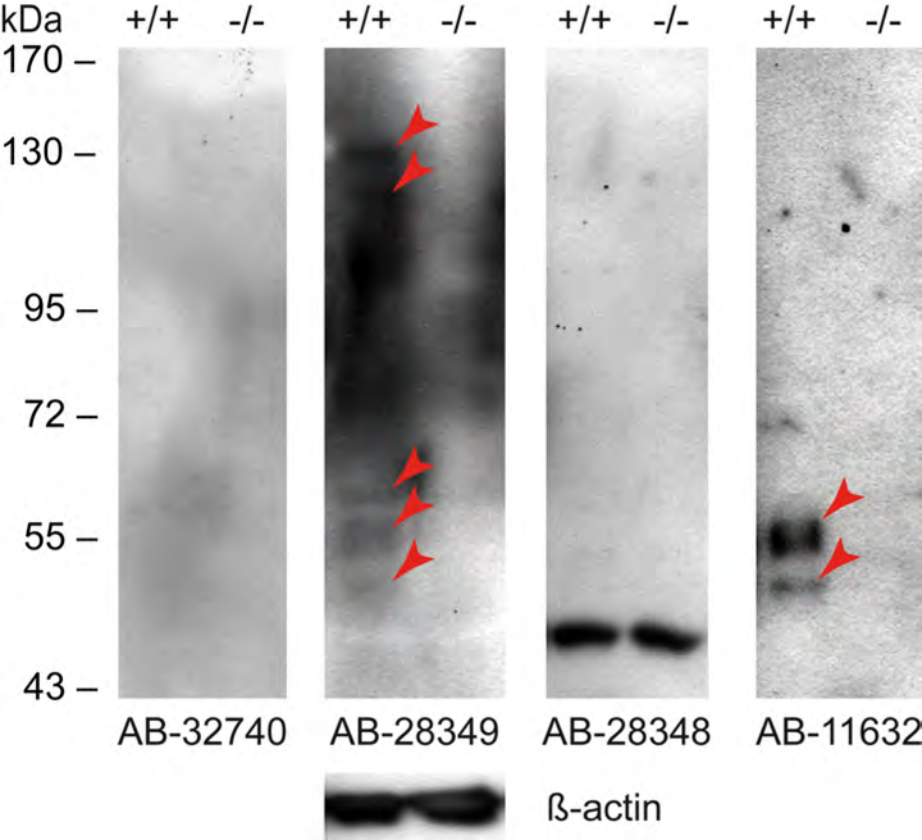

Supplement: Figure S1 — Western blot analysis of wildtype and Jmjd6 knockout MEFs using different anti-Jmjd6 antibodies. This panel shows western blots with total cell lysates from wildtype and Jmjd6-KO MEFs using four different antibodies against Jmjd6. AB-32740, AB-28349, AB-28348, and AB-11632 were evaluated. Red arrowheads mark bands not present in Jmjd6-knockout lanes. All lanes contain the same amount of protein and are from the same sample. Beta-actin served as a loading control for equal amounts of protein on the gel. (1.57 MB PDF) [file pone.0013769.s001.pdf]

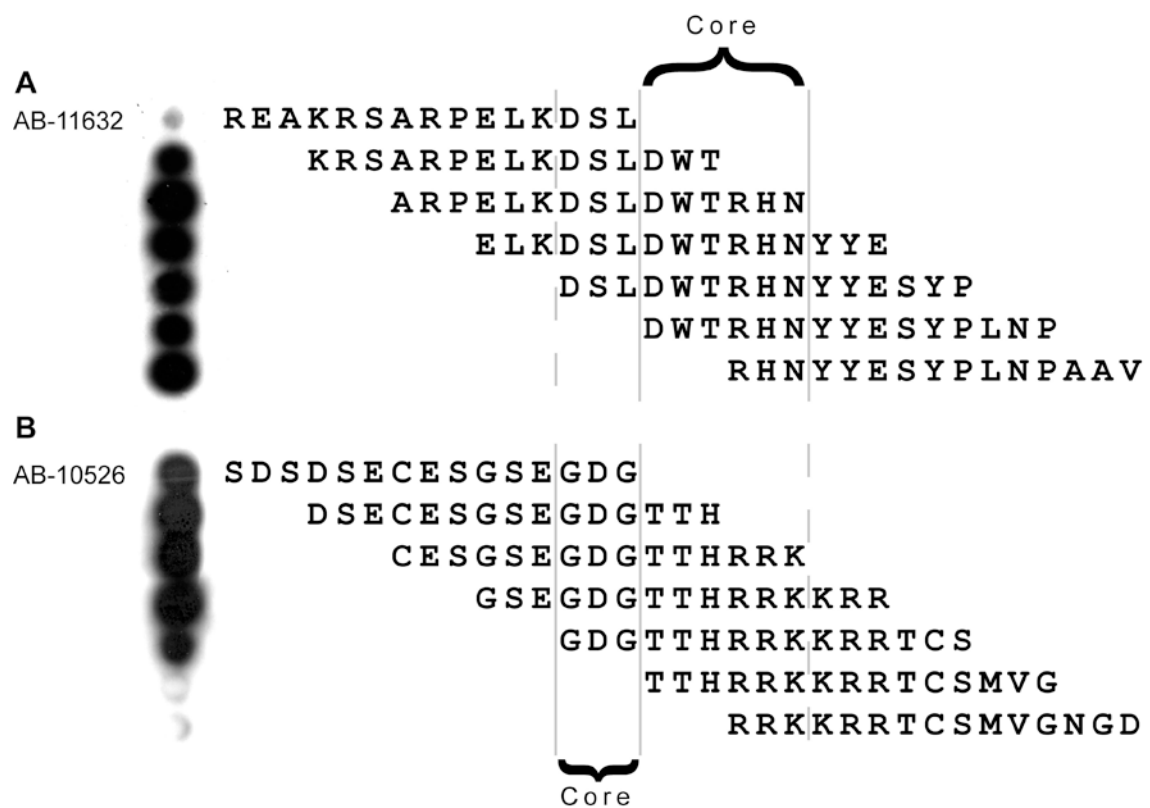

Supplement: Figure S2 — Epitope-mapping of AB-11632 and AB-10526. A spot membrane covering the full length Jmjd6 protein in fifteenmers, each shifted by three amino acids, was incubated with AB-11632 and AB-10526. (A) AB-11632 binds to seven fifteenmers and six thereof show high intensity. The core epitope covers the amino acids DWTRHN. (B) AB-10526 binds to seven fifteenmers and five thereof show high intensity. The core epitope covers the amino acids GDG. (0.07 MB PDF) [file pone.0013769.s002.pdf]

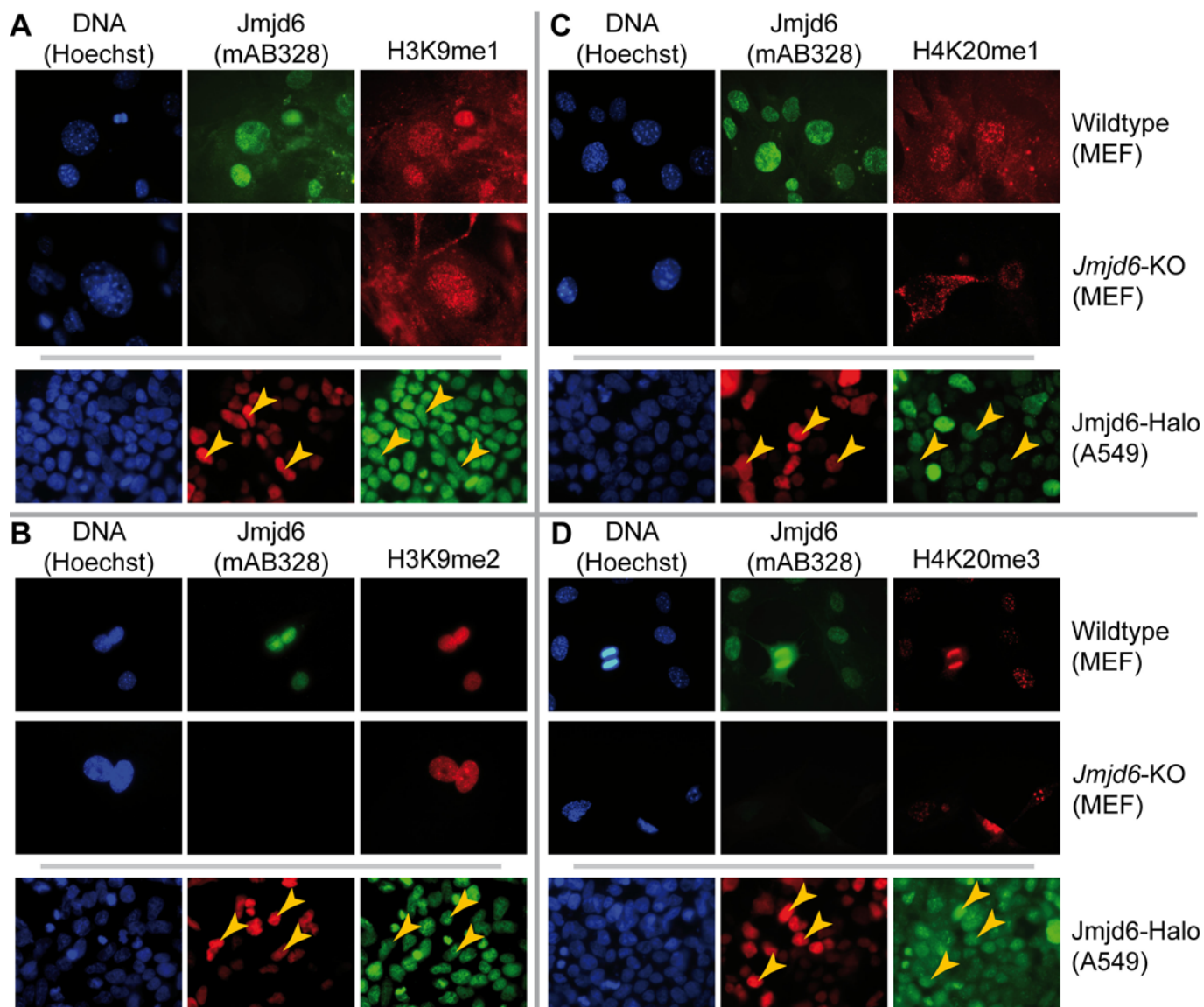

Supplement: Figure S4 — Single cell analysis of H3K9 and H4K20 methylation effects caused by Jmjd6 deficiency or overexpression. The top two rows show immunofluorescence images of Hoechst DNA stain (blue), Jmjd6 (green), and a histone lysine methylation state specific antibody (red) from wildtype and Jmjd6-KO MEFs (from left to right, respectively). The bottom row shows immunofluorescence images of Hoechst DNA stain (blue), Jmjd6 (red), and a histone lysine methylation state specific antibody (green) in Jmjd6-Halo overexpressing A549 cells (from left to right, respectively). Yellow arrowheads indicate transfected cells. Figure (A) shows results for H3K9me1, (B) for H3K9me2, (C) for H4K20me1, and (D) for H4K20me3. All experiments shown were performed at least at least three times with similar results. (0.76 MB PDF) [file pone.0013769.s004.pdf]

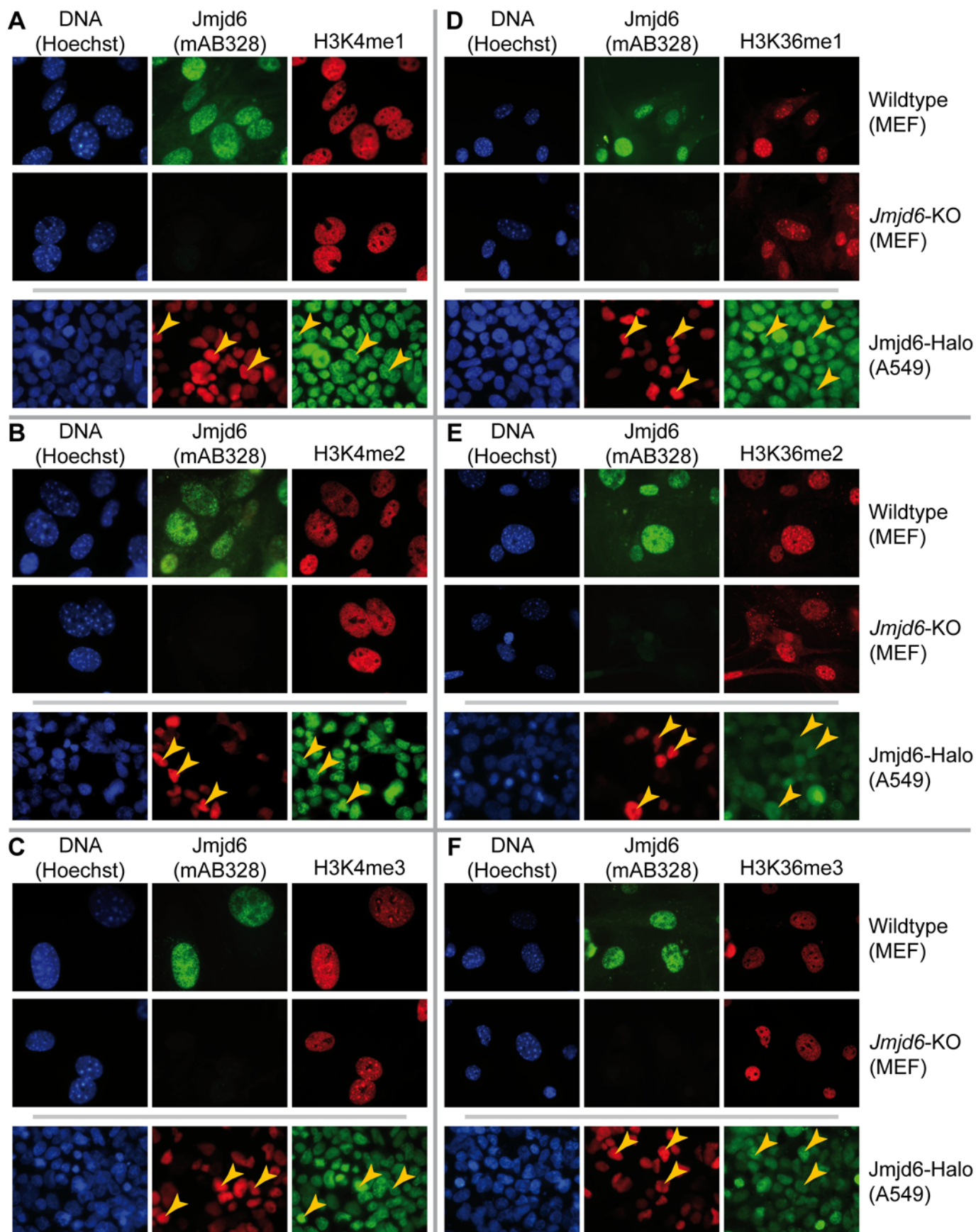

Supplement: Figure S5 — Immunofluorescence analysis of H3K4 and H3K36 methylation effects caused by Jmjd6 deficiency or overexpression. The top two rows show immunofluorescence images of Hoechst DNA stain (blue), Jmjd6 (green), and a histone lysine methylation state specific antibody (red) from wildtype and Jmjd6-KO MEFs (from left to right, respectively). The bottom row shows immunofluorescence images of Hoechst DNA stain (blue), Jmjd6 (red), and a histone lysine methylation state specific antibody (green) in Jmjd6-Halo overexpressing A549 cells (from left to right, respectively). Yellow arrowheads indicate transfected cells. Figure (A) shows results for H3K4me1, (B) for H3K4me2, (C) for H3K4me3, (D) for H3K36me1, (E) for H3K36me2, and (F) for H3K36me3. The experiments were performed at least three times with similar results. (1.22 MB PDF) [file pone.0013769.s005.pdf]

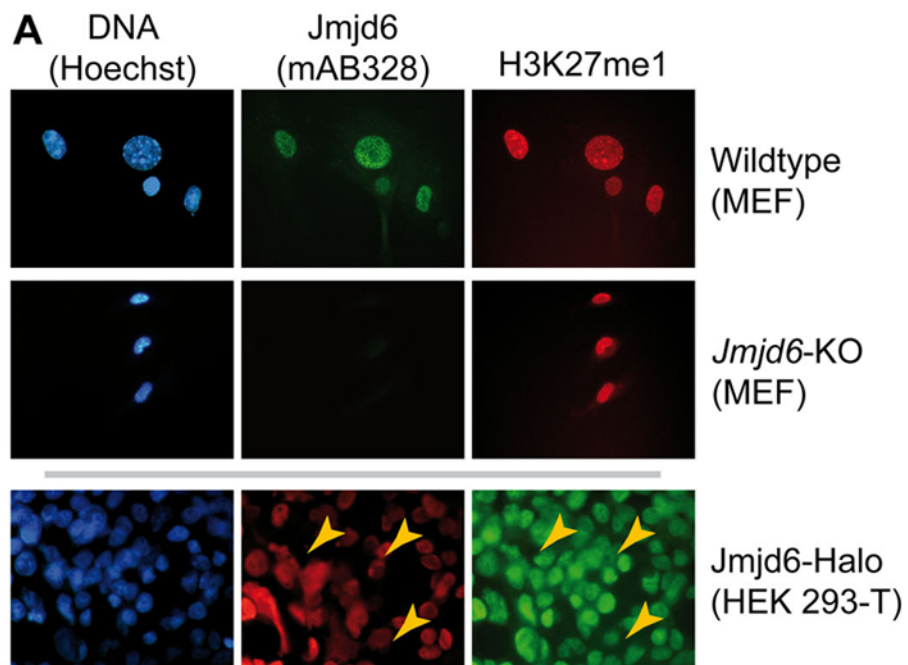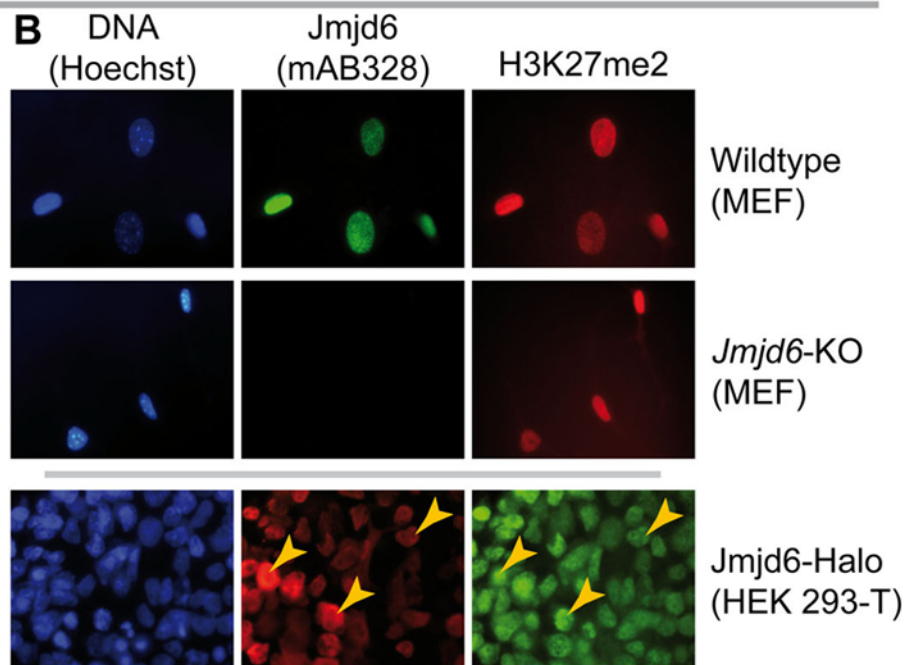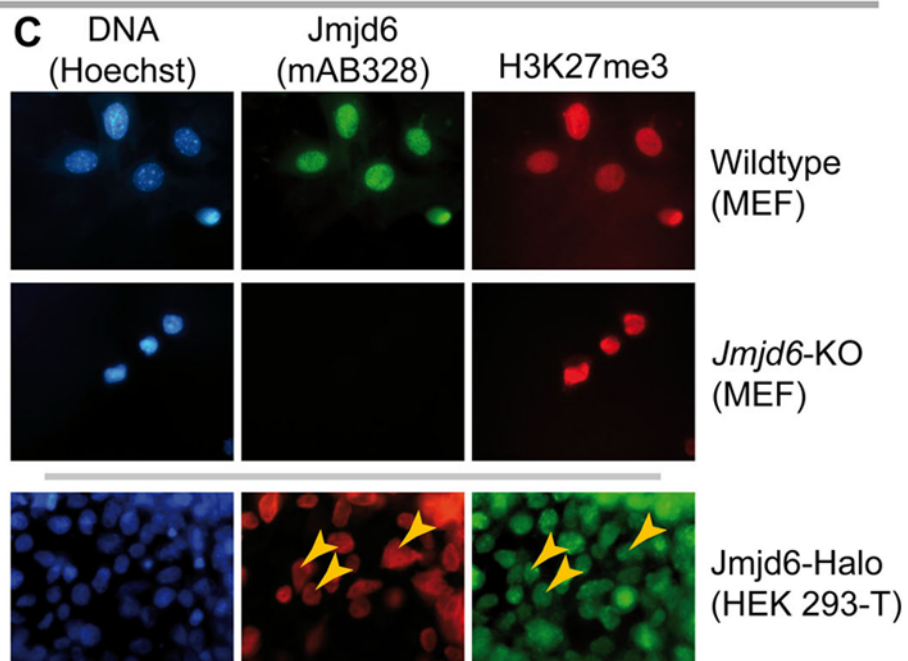

Supplement: Figure S6 — Immunofluorescence analysis of H3K27 methylation effects caused by Jmjd6 deficiency or overexpression. The top two rows show immunofluorescence images of Hoechst DNA stain (blue), Jmjd6 (green), and a histone lysine methylation state specific antibody (red) from wildtype and Jmjd6-KO MEFs (from left to right, respectively). The bottom row shows immunofluorescence images of Hoechst DNA stain (blue), Jmjd6 (red), and a histone lysine methylation state specific antibody (green) in Jmjd6-Halo overexpressing HEK 293-T cells (from left to right, respectively). Yellow arrowheads indicate transfected cells. Figure (A) shows results for H3K27me1, (B) for H3K27me2, and (C) for H3K27me3. The experiments were performed three times with similar results. (0.85 MB PDF) [file pone.0013769.s006.pdf]
