# Supplementary material for: Analysis of Jmjd6 Cellular Localization and Testing for Its Involvement in Histone Demethylation
Source: PLoS One. 2010 Oct 29;5(10):e13769. doi: 10.1371/journal.pone.0013769 (PMC2966431; doi:10.1371/journal.pone.0013769)
Supplement: Figure S3 — Jmjd6 staining pattern in different cell lines and Jmjd6 distribution over time after releasing A549 cells from a G0-block. (A) Comparison of Jmjd6 nuclear expression pattern between murine MEFs and the human cell lines A549 and HEK 293-T. Cells were stained with Hoechst DNA stain (left panel) and for Jmjd6 using the anti-Jmjd6 mAB328 antibody (right panel). (B) Vertical image pairs correspond to a time point 6 h, 24 h, 48 h, or 72 h after releasing A549 cells from a G0-block. The large images show Jmjd6 distribution in green, the small insets the corresponding Hoechst DNA stain in blue. Red arrowheads indicate nuclei with deviant intranuclear Jmjd6 distribution. Shown is one representative experiments of at least at least three performed. (4.22 MB PDF) [file pone.0013769.s003.pdf]

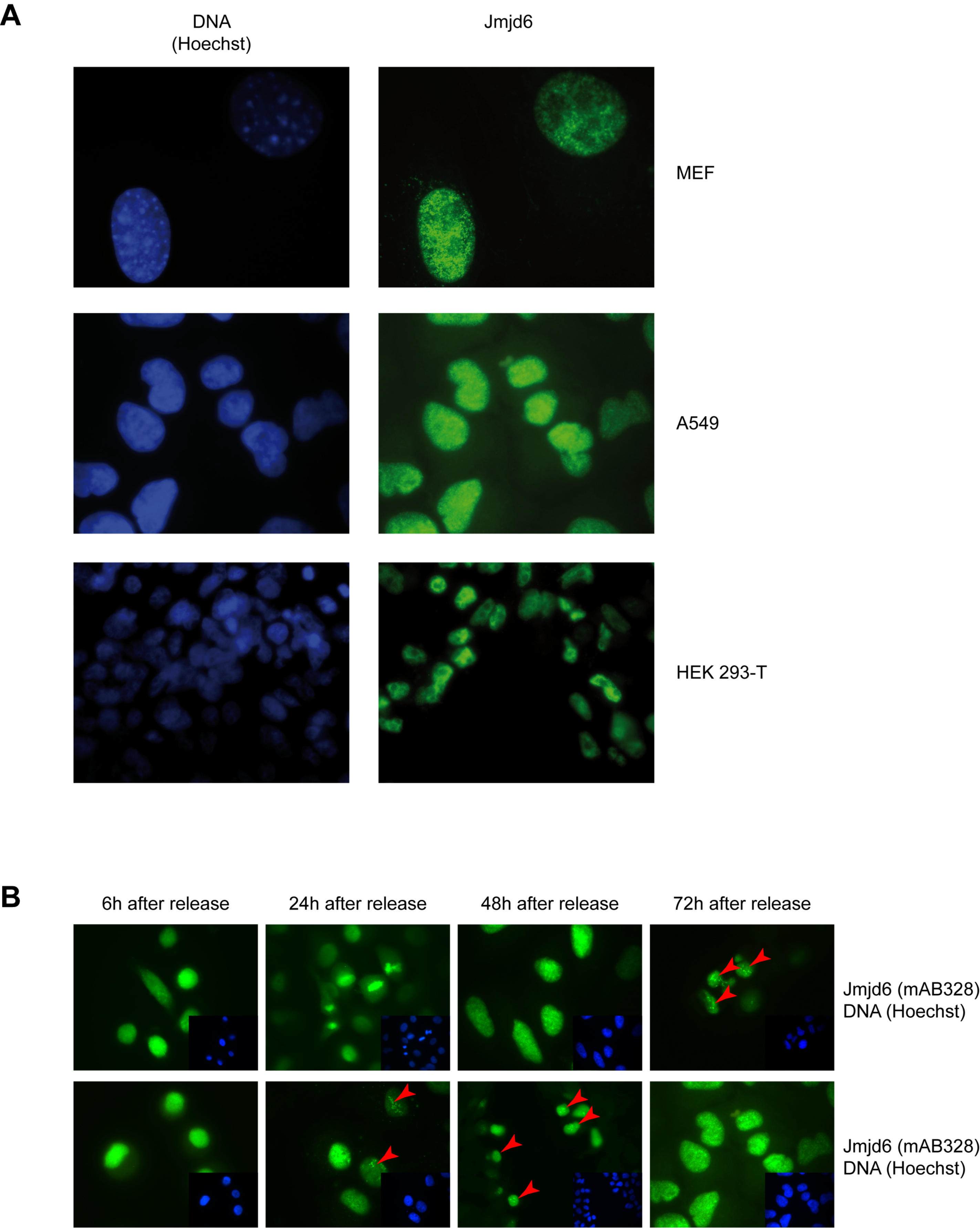

A
DNA (Hoechst)
Jmjd6
MEF
A549
HEK 293-T
B
6h after release
24h after release
48h after release
72h after release
Jmjd6 (mAB328)
DNA (Hoechst)
Jmjd6 (mAB328)
DNA (Hoechst)
