# Supplementary material for: Analysis of Jmjd6 Cellular Localization and Testing for Its Involvement in Histone Demethylation
Source: PLoS One. 2010 Oct 29;5(10):e13769. doi: 10.1371/journal.pone.0013769 (PMC2966431; doi:10.1371/journal.pone.0013769)
Supplement: Table S1 — Primer pairs used for cloning of Jmjd6 fusion/reporter proteins. Sequences of primers used for the cloning of Jmjd6 fusion, reporter and deletion constructs. (0.04 MB PDF) [file pone.0013769.s007.pdf]

Supplementary Table 1: Primer pairs used for cloning of Jmjd6 fusion / reporter proteins

No.	Product	Primer pair	Size (bp)
1	Jmjd6	aggctagcatgaaccacaagagcaagaagcgc<br>tcggatcccctggaggagctgcgctctttgc	1225
2	Jmjd6<br>(AxA)	actcgagatgaaccacaagagcaagaagcgcatcc<br>tccaggcactggtccccagaggggcgatggcaatcccag	596
3	YFP	tttcgctagcatgtcgacggatccgcggccgctcatggtgagcaagggcg aggagc<br>ctcgagttacttgtacagctcgtccatgccga	760
4	Jmjd6<br>F1/R1	atgtcgacgaaccacaagagcaagaagcgc<br>cagcggccgcgggtgttcgccatagctgctatc	438
5	Jmjd6<br>F1/R3	atgtcgacgaaccacaagagcaagaagcgc<br>cagcggccgcggaaagttctgggtgatggcaatgg	883
6	Jmjd6<br>F1/R4	atgtcgacgaaccacaagagcaagaagcgc<br>cagcggccgcggtctccctcttaccgtcttgtg	933
7	Jmjd6<br>F1/R5	atgtcgacgaaccacaagagcaagaagcgc<br>cagcggccgcggcctggaggagctgcgctc	1227
8	Jmjd6<br>F2/R4	atgtcgacgcccaaaagaaggaaacttttg<br>cagcggccgcggtctccctcttaccgtcttgtg	934
9	Jmjd6<br>F2/R5	atgtcgacgcccaaaagaaggaaacttttg<br>cagcggccgcggcctggaggagctgcgctc	810
10	Jmjd6<br>F3/R5	atgtcgacgtttgtaccagggggctggtgg<br>cagcggccgcggcctggaggagctgcgctc	435
11	Jmjd6<br>F5/R5	atgtcgacgccaaagttatcaaggaagtgg<br>cagcggccgcggcctggaggagctgcgctc	315
