# Supplementary material for: Analysis of Jmjd6 Cellular Localization and Testing for Its Involvement in Histone Demethylation
Source: PLoS One. 2010 Oct 29;5(10):e13769. doi: 10.1371/journal.pone.0013769 (PMC2966431; doi:10.1371/journal.pone.0013769)
Supplement: Table S2 — Antibodies used in Western blotting and immunofluorescence imaging. List of antibodies used for western blotting and immunocytochemistry. (0.06 MB PDF) [file pone.0013769.s008.pdf]

Supplementary Table 2: Antibodies used in Western blotting and immunofluorescence imaging

Target	Name	Dilution (Western)	Dilution (IF)	Clone No.	Distributor
ß-actin	anti-ß-actin	1:5000	–	ab6276	abcam
HaloTag	anti-HaloTag	1:1000	1:500	G9281	Promega
Histone H3K4me1	H3K4me1	1:500	1:500	ab8895	abcam
Histone H3K4me2	H3K4me2	1:500	1:500	ab7766	abcam
Histone H3K4me3	H3K4me3	1:500	1:500	ab8580	abcam
Histone H3K9me1	H3K9me1	1:500	1:100	ab9045	abcam
Histone H3K9me2	H3K9me2	1:500	1:500	ab7312	abcam
Histone H3K9me3	H3K9me3	1:500	–	ab8898	abcam
Histone H3K27me1	H3K27me1	1:500	1:500	07-448	upstate
Histone H3K27me2	H3K27me2	1:500	1:500	07-421	upstate
Histone H3K27me3	H3K27me3	1:500	1:500	07-449	upstate
Histone H3K36me1	H3K36me1	1:500	1:500	ab9048	abcam
Histone H3K36me2	H3K36me2	1:500	1:500	07-369	upstate
Histone H3K36me3	H3K36me3	1:500	1:500	ab9050	abcam
Histone H4K20me1	H4K20me1	1:500	1:100	ab9051	abcam
Histone H4K20me2	H4K20me2	1:500	–	ab9052	abcam
Histone H4K20me3	H4K20me3	1:500	1:500	ab9053	abcam
Jmjd6	mAB328	–	1:500	mAB328C 6D11	–
Jmjd6	AB-10526	1:7500	1:200	ab10526	abcam
Jmjd6	AB-11632	1:1000	1:200	sc-11632	Santa Cruz
Jmjd6	AB-32740	1:300	1:200	sc-32740	Santa Cruz
Jmjd6	AB-28349	1:300	1:200	sc-28349	Santa Cruz
Jmjd6	AB-28348	1:300	1:200	sc-28348	Santa Cruz
GFP	AB-290	1:2000	-	ab290	abcam
anti-mouse Alexa 488	–	–	1:200	A-11017	Invitrogen
anti-mouse Alexa 568	–	–	1:200	A-11019	Invitrogen
anti-rabbit Alexa 488	–	–	1:200	A-11070	Invitrogen
anti-rabbit Alexa 568	–	–	1:200	A-21069	Invitrogen
anti-mouse HRP	–	–	1:10000	ab6789	abcam
anti-rabbit HRP	–	–	1:10000	ab6721	abcam
anti-goat HRP	–	–	1:10000	sc-2922	Santa Cruz
